# Identifying and predicting heat stress events for grazing dairy cows using rumen temperature boluses

**DOI:** 10.3168/jdsc.2023-0482

**Published:** 2024-01-15

**Authors:** S.J.R. Woodward, J.P. Edwards, K.J. Verhoek, J.G. Jago

**Affiliations:** 1 DairyNZ Ltd., Hamilton 3240, New Zealand; 2 DairyNZ Ltd., Lincoln University, Lincoln 7647, New Zealand

## Abstract

Heat stress events in dairy cows are associated with behavioral and physiological changes such as seeking shade, increased respiration rate and body temperature, reduced milk production, and psychological distress. Knowledge of the relationship between weather and animal responses to heat stress enables automated alerts using forecast weather, aiding early provision of shade or other mitigation practices. While numerous heat stress indices for cattle have been developed, these have limitations for cows exposed to wind and solar radiation (i.e., predominantly grazing outdoors or managed on pasture). To develop a predictive model for heat stress events in pasture-based dairy systems, rumen temperature data from smaXtec (smaXtec animal care GmbH, Graz, Austria) rumen boluses in 443 cows on 3 dairy farms in Northland, New Zealand, were used to identify heat stress events and these were matched with automated weather station data collected on or near the farm. Heat stress rate (HSR) was defined as the percentage of cows within an age-breed group having a rumen temperature greater than 3 standard deviations above an individual cow's mean and heat stress events were defined as HSR >25%. Single and multiple linear regression models, including published heat stress indices, were generally able to predict a high proportion of heat stress events (sensitivity 34%–68%), but were insufficiently discriminating, predicting also a high number of false positives (precision only 9%–27%). A machine learning algorithm, cubist, was the best performing model, predicting 79% of heat stress events with a precision of 52% for this dataset. Our proof-of-concept study demonstrates the potential of this approach, using climate data to predict and forecast heat stress events in pasture-based dairy systems. Further work should test the cubist model using independent data, refine dataset construction, investigate the value of including known animal variables such as cow age or breed, and incorporate other measures of heat stress such as respiration rate.

## Highlights

• Rumen bolus containing a temperature sensor allows for high-resolution data capture.
• A large dataset from grazing dairy cows was captured with this technology.
• Using rumen temperature and machine learning has promise for predicting heat stress.
• Further testing is required to confirm the robustness of this outcome.

## Graphical Abstract

**Figure:**
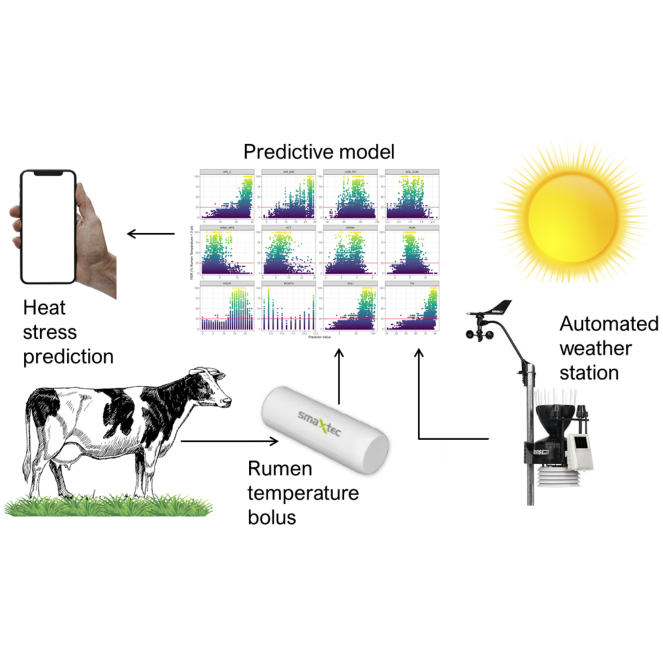
**Summary** We present a new approach to developing predictive models of heat stress risk for grazing dairy herds. This proof-of-concept study demonstrates the potential of using a machine learning algorithm to predict heat stress events defined using rumen temperature. The approach has been enabled by animal sensors collecting large amounts of data on individual cows. The success of this approach is encouraging, as the scale and variable nature of farming outdoors in temperate climates has, to date, limited the development of our understanding and management of thermal stress among dairy cattle in these systems. To develop this approach further will require testing with independent datasets from a wider range of environments alongside model tuning.

Weather conditions in summer can lead to heat stress in dairy cattle with negative impacts on milk production and welfare, particularly in pasture-based dairy systems where there is less ability to manage the environment than in indoor systems. Heat stress risk is expected to be exacerbated as daily temperatures continue to rise and extreme hot weather events become more frequent (Jago et al., 2023). The ability to identify or predict the onset of herd or group level heat stress in dairy cows could enable operational management decisions (such as timing of milking or choice of paddock) to reduce its impact on-farm as well as to allow strategic management planning (such as changing milking frequency, breeding choices, tree planting, and installation of portable shade structures) in response to the potential impact of climate change.

Previous research has investigated the relationship between weather conditions and respiration rate for grazing dairy cows (Bryant et al., 2023). Manual observation of animal respiration rate is the gold standard indicator for heat stress response; however, its collection is labor intensive, prone to operator error, performed only at discrete points in time, and not scalable to large numbers of animals, or in situations of high stocking density (Wijffels et al., 2021). This limits the ability to collect a sufficiently large dataset across many environments for training and validation of predictive models. For example, the study of Bryant et al. (2023) used observations from only a single geographical region. Therefore, the use of automation would be beneficial to enhance the understanding of the relationship between animal responses to climatic conditions that could cause heat stress in a grazing environment.

Large datasets collected by sensor technologies provide an alternative approach to train models for predicting heat stress risk and determine the efficacy of management mitigations when heat stress conditions exist. One sensor-based approach is to use accelerometer data to predict heavy breathing, and the proportion of cows breathing heavily in a group has been shown to mirror changes in vaginal temperature (Bar et al., 2019). Similarly, rumen temperature has been shown to be correlated with rectal temperature (Bewley et al., 2008; Boehmer, 2015) and has been used to evaluate the effects of heat stress (Lees et al., 2018). Once inserted, rumen temperature boluses record rumen temperature continuously, and do not require visual assessment of placement, so require less work for data collection over long periods than rectal or vaginal temperature loggers. However, the applicability of rumen temperature boluses to indicate heat stress in pasture-based dairy systems, and the weather conditions that drive it, is relatively unknown. The objective of this study was to explore the feasibility of identifying and predicting heat stress events in grazing dairy cows from automatically monitored weather conditions. We hypothesized that rumen bolus sensor data could be used to identify heat stress events and automated weather data could be used to develop a model to predict these events.

The study used “smaXtec” (smaXtec animal care GmbH, Graz, Austria) rumen bolus data from 443 cows located on 3 farms near Dargaville (Northland, New Zealand), from the period of January 1, 2021, to June 7, 2023. Farm 1 had a subset of 192 cows that had smaXtec boluses from a herd of ~230 cows. Farm 2, located ~17 km north-northeast from farm 1, had a subset of 49 cows with boluses from a herd of ~400 cows. Farm 3, located ~4 km east-northeast from farm 2, had rumen temperature data from 259 unique cows from a herd of ~180 cows. On farms 1 and 2, no new boluses were added to replacement animals entering the herd, which differed from farm 3 where animals that entered the herd to replace culls, sales, and deaths received boluses. At farms 2 and 3, cows were always milked once per day in the morning. No specific heat stress mitigations (e.g., provision of sprinklers) were used on any of the farms. Institutional Animal Care and Use Committee or equivalent approval was not obtained because data were already recorded by the farms for their own use.

Data obtained from the smaXtec bolus included rumen temperature (“temp_without_drink_cycles”), which is the raw rumen temperature corrected by the manufacturer's proprietary algorithm for the effects of drinking events, as well as additional interpretive variables activity (“act”), rumination (“rum_index”), and drinking events (“drink_cycles_v2”), stored at both 10-min and 1-h resolution. The current study focused on prediction using rumen temperature. The correction algorithm removed short-term drops in rumen temperature presumed to be caused by drinking events; however, the details of the proprietary algorithm are unknown.

Weather data for the same period were obtained from 2 Davis Wireless Vantage Pro2 Plus weather stations (Davis Instruments, Hayward, CA). One station was located at farm 1, the other was located at farm 2 until it was moved, for reasons relating to another project, to farm 3 on January 7, 2023. Due to the proximity (4 km) of farms 2 and 3, the weather was assumed to be the same at each site. Data were accessed via the WeatherLink v2 API (sensor numbers 43, 52, 53, 56, 242, 243, and 504). Weather data were available at 15-min intervals and included air temperature (°C), humidity (%), solar radiation (MJ·m^−2^·h^−1^), rainfall (mm), and wind speed (m·s^−1^). Additional metrics, such as daily minimum air temperature, cumulative solar radiation, temperature-humidity index (**THI**; Thom, 1959), and grazing heat load index (**GHLI**; Bryant et al., 2023) were calculated from these, noting we were advised by the authors that the GHLI equation requires wind speed in km·h^−1^, not m·s^−1^ as published.

Both datasets were stored in a Snowflake database (Snowflake Inc., Bozeman, MT). Using the dbplyr package in R (R Core Team, 2023; Wickham et al., 2023), the weather data on the hour were joined to the hourly smaXtec bolus data for the 3 farms. The resulting table of 5.28 million rows (cows × hours) was downloaded and saved as a parquet file (size 113 Mb). Variables of interest (as listed above) were examined, and spurious values were replaced with “not available.” Cows with evidently implausible activity (1 cow), drinking (1 cow), or rumen temperature (6 cows) data were assumed to have faulty sensors and their data were removed.

Four main steps were undertaken to analyze the data. (1) A suitable indicator of heat stress incidence was proposed and heat stress events were identified. (2) The data were balanced between heat-stressed and non-heat-stressed states (Branco et al., 2016). (3) Several regression models were tested for predicting the heat stress rate (**HSR**) from weather data. (4) These models were compared with existing heat load indexes for the prediction of heat stress events, particularly the GHLI (Bryant et al., 2023).

Because we lacked respiration rate or panting data, heat stress incidence was defined using rumen temperature, which has been previously linked to thermal stress in dairy cattle (Donkersloot et al., 2017; Levit et al., 2021). The cumulative distribution of rumen temperature (hourly; adjusted for drinking) of individual cows varied in mean (gray vertical lines) and SD (Figure 1a). This highlighted that a fixed temperature threshold (e.g., 39.5°C, Liu et al., 2019; Levit et al., 2021) was unlikely to indicate heat stress across all animals. The smaXtec rumen temperature for each cow was therefore scaled so that all cows had a common mean and SD (Figure 1b). This provided the basis for a proposed HSR metric for a group or herd, which was defined as the percentage of cows with scaled rumen temperature >3 SD above the mean (indicated by the vertical line in Figure 1b). Three SD from the mean is often used to indicate “extreme” events (Grafarend, 2006) and was proposed as a proof of concept here.

**Figure 1:**
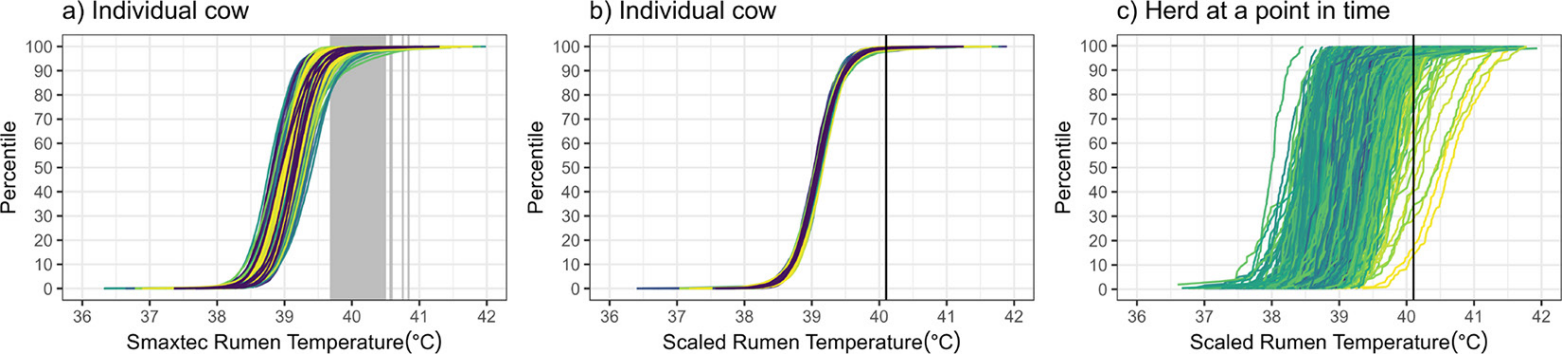
Variation in smaXtec (smaXtec animal care GmbH) rumen temperature (a) for individual cows, (b) after scaling to a common mean, and (c) between cows in a herd at a point in time. Vertical lines in each facet indicate 3 SD above the mean; note this appears as shading in (a). Traces in (a) and (b) are colored by cow number and in (c) are colored by air temperature ranging from 0°C (blue) to 30°C (yellow).

For each hour, the individual cow data were grouped by farm, cow age (≤4, 4–8, 8–12, or >12 yr), and cow breed (≤4, 4–8, 8–12, or >12 parts Jersey out of 16, with the other parts principally being Friesian), and HSR was calculated for each group. Groups with small numbers of cows (<5) were excluded to reduce noise.

In this study we chose to predict HSR from weather variables alone, so that in the future the output could be used to provide a regional forecast of expected heat stress events (proposed to occur when HSR exceeded 25%, see below). The key weather variables used were air temperature (**AIR_C**; °C), daily minimum air temperature (**AIR_MIN**; °C), daily cumulative mean solar radiation (**SOL_CUM**; MJ·m^−2^·h^−1^), relative humidity (**HUM_PC**; %), and windspeed (**WIND_MPS**; m·s^−1^). Figure 2 shows correlations between these weather variables and HSR. Other cow behavior variables (activity, **ACT**; rumination, **RUM**; drinking, **DRINK**) are also shown in Figure 2, as are the hour of the day (**HOUR**) and the month of the year (**MONTH**). Also shown are the THI of Thom (1959) and the more recently developed GHLI of Bryant et al. (2023), which is based on temperature, wind speed, and solar radiation. THI is in units of temperature (°F) and GHLI is in units of respiration rate (min^−1^).

**Figure 2:**
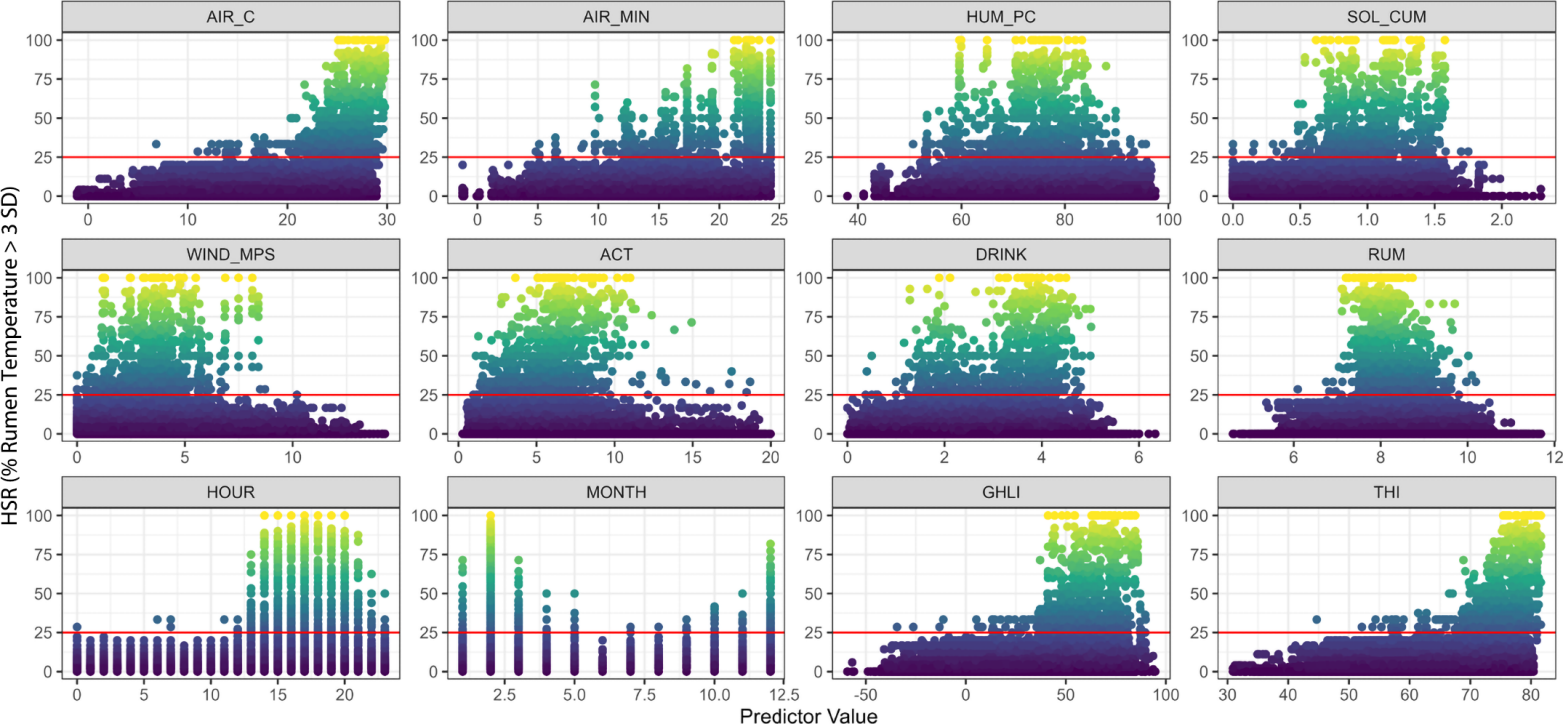
Relationship between heat stress rate (HSR, proportion of cows with rumen temperature >3 SD above the mean) and weather variables (air temperature, AIR_C, °C; minimum daily air temperature, AIR_MIN, °C; humidity, HUM_PC, %; cumulative mean solar radiation, SOL_CUM, MJ m^−2^ h^−1^; wind speed, WIND_MPS, m s^−1^), related cow behavior (activity, ACT; rumination, RUM, h d^−1^; drinking, DRINK, n d^−1^), temporal patterns (HOUR, MONTH), and climate indices (temperature-humidity index [THI], grazing heat load index [GHLI]). Colors range from 0% (blue) to 100% (yellow). The red line shows the proposed threshold (HSR >25%) indicating heat stress events.

Based on these data, a threshold at HSR >25% was proposed as a suitable indicator of heat stress events, as values of HSR >0% and <25% can occur under any conditions (e.g., at nighttime), whereas HSR >25% observations generally matched expected heat stress conditions (high AIR_C, high AIR_MIN, high HUM_PC, high SOL_CUM, low WIND_MPS, low ACT, high DRINK, low RUM, afternoon HOUR, summer MONTH). On this basis, heat stress events were detected on 27, 23, and 35 d at farms 1, 2, and 3 (out of 324, 330, and 330 d, respectively).

Models based on severely imbalanced training data tend to perform poorly when predicting the minority cases that are of primary interest (in this case, heat stress events; Branco et al., 2016). To avoid this, hours with air temperature ≤20°C were first excluded because heat stress rarely occurs under these conditions (Bohmanova et al., 2007; Bryant et al., 2007). The remaining data were then resampled (Branco et al., 2016) to achieve similar numbers of positive (defined as HSR >25%) and negative (defined as HSR ≤25%) data rows. The probability of retaining a data row was *P* = 100% when HSR was >25% (806/806 rows), *P* = 5% + (100% − 5%) × HSR ÷ 25% when HSR was in the range 0–25% (1,478/4,121 rows), and *P* = 5% when HSR was 0% (2,433/48,022 rows). Resampling reduced the dataset from 52,949 to 4,717 rows. Although it would have been desirable to hold back some positive data rows for model testing, this would have resulted in too small of a training set.

A range of models was tested for prediction of HSR and heat stress events (HSR >25%). These included simple linear regression (using variables such as AIR_C, THI, or GHLI), multiple linear regression (lm), generalized additive models (gam; Wood, 2011), and machine learning models from the “caret” package in R (Kuhn, 2008), specifically random forest (rf), cubist, gradient boosted machine (gbm), support vector machine with radial basis (svmRadial), and k-nearest neighbors (knn), trained using the default settings. Three models were chosen for presentation here, and comparison with the GHLI. The models were trained using the 4,717 training cases, then validated against the entire dataset of 52,949 cases (which were predominantly negative cases).

Figure 3a shows the model predictions of HSR over the entire dataset, compared with the proposed threshold of 25% (red lines). Each data value (y-axis) is plotted against the corresponding model prediction (x-axis). For each model, the following statistics are reported (Branco et al., 2016): the coefficient of determination (“Rsq”; the proportion of variance in the data explained by the model), accuracy (“Acc”; the proportion of correct predictions), sensitivity (“Sen”; the proportion of positive events that the model correctly predicted), precision (“Pre”; the proportion of predicted events that were, in fact, positive events according to the data; also known as the positive predictive value), and the F_1_ score, which is a summary of the model's ability to predict positive cases. The formulas for these metrics are Rsq = 1 – ∑*_i_*(*HSR_model,i_* – *HSR_data,i_*)^2^ ÷ $\Sigma_{i}(\bar{HSR_{data}}$ – *HSR_data,i_*)^2^, Acc = (TP + TN) ÷ (TP + TN + FP + FN), Sen = TP ÷ (TP + FN), Pre = TP ÷ (TP + FP), F_1_ = (2 × Sen × Pre) ÷ (Sen + Pre), where *HSR_model,i_* are the model predictions corresponding to the data *HSR_data,i_* and $\bar{HSR_{data}}$ is the mean value of the HSR calculated from the data. TP, TN, FP, and FN are the number of true positives (HSR >25%), true negatives, false positives, and false negatives, respectively. Figure 3b shows the variable importance assessed using the “iml” package in R (Molnar et al., 2018), which is the increase in prediction error that occurs when each variable is removed. While the data points themselves are not without error, the results in Figure 3 clearly illustrate the improvement in predictive ability as model sophistication increased.

**Figure 3:**
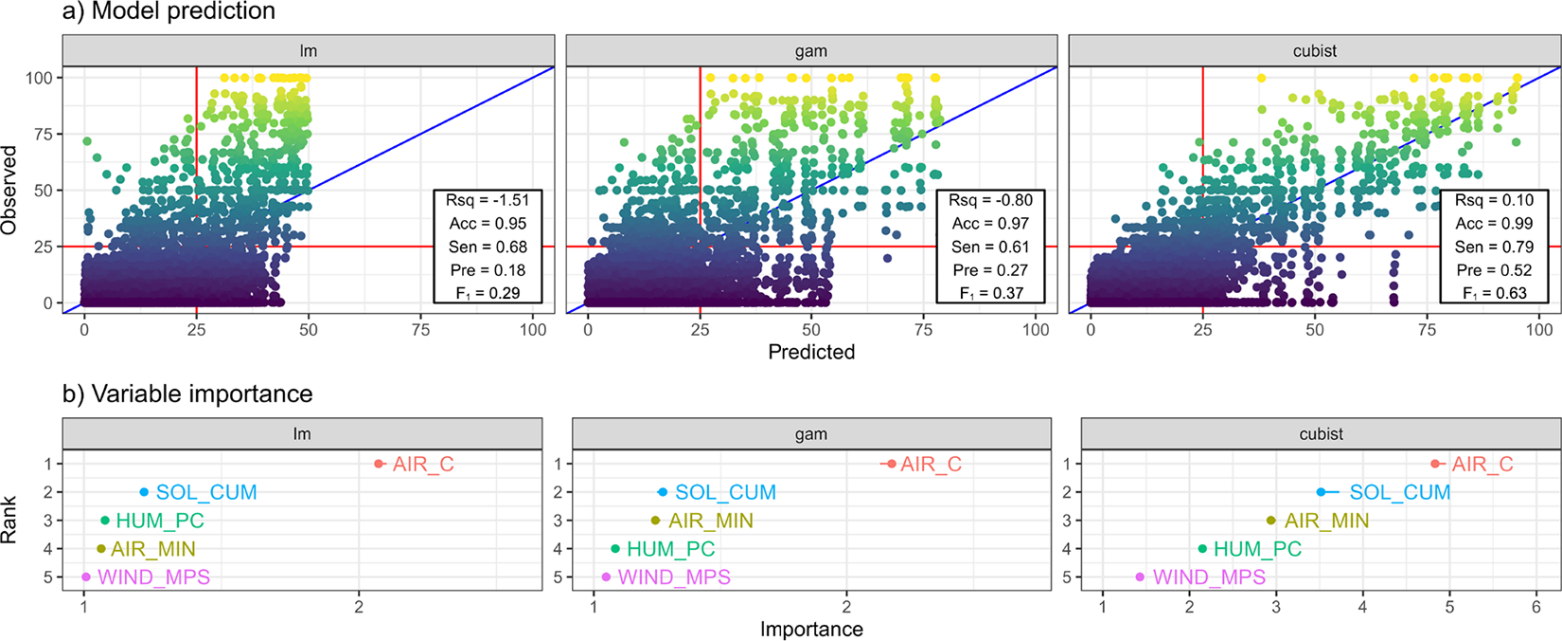
(a) Model predictions (lm, gam, cubist) based on the weather variables air temperature (AIR_C, °C), minimum daily air temperature (AIR_MIN, °C), humidity (HUM_PC, %), cumulative mean solar radiation (SOL_CUM, MJ·m^−2^·h^−1^), and wind speed (WIND_MPS, m·s^−1^) of heat stress rate (HSR; proportion of cows with rumen temperature >3 SD above the mean) compared with observed values for the full dataset, and (b) variable importance ranking in each model. Red lines show the proposed threshold (HSR >25%) indicating heat stress events, and the blue line is the 1:1 line. Rsq = proportion of variance in the data explained by the model; Acc = proportion of correct predictions; Sen = proportion of positive events that the model predicted; Pre = proportion of model predicted events that were, in fact, positive according to the data; F_1_ = summary of the model's ability to predict positive cases [F_1_ = 2 × Sen × Pre ÷ (Sen + Pre)]. For comparison, the grazing heat load index (GHLI) and threshold of Bryant et al. (2023) produced values of Acc = 0.94, Sen = 0.34, Pre = 0.09, and F_1_ = 0.14.

The simple GHLI model (a linear model of cow respiration rate based on air temperature, windspeed, and solar radiation) is proposed to predict heat stress events in grazing dairy cows, as indicated by marked changes in panting and drooling, when its value is 70 or more (Bryant et al., 2023). When applied to the current dataset, the GHLI had a sensitivity of 34% and a precision of only 9%, indicating a high proportion of false positives (Figure 3). Allowing for the different definitions of heat stress, this result indicates that the GHLI predicted several events where there was limited corresponding increase in rumen temperature (HSR ≤25%), indicating a need for further understanding about respiration rate as an indicator of when animals experience heat stress and how heat stress should be defined. A multivariate linear model (lm) using AIR_C, AIR_MIN, SOL_CUM, HUM_PC, and WIND_MPS performed better. An additive nonlinear model using the same variables (gam) improved on this further. However, only machine learning models (e.g., cubist), which could fully exploit the structure of the data, gave predictions at an acceptable level of sensitivity and precision (i.e., detecting a high proportion of events, but without as many false positives; Figure 3).

Training Rsq and accuracy were greater than 0 and 0.50, respectively, for all models. However, when the models were tested by predicting against the full dataset, it became clear that Rsq and accuracy were not useful metrics of predictive performance. The large number of negative cases in the full dataset resulted in low or negative Rsq values and misleadingly high values of accuracy. By comparison, sensitivity, precision, and F_1_ gave more useful metrics of predictive performance. Only the performance of the cubist model was high enough that it may be useful for practical application (random forest “rf” performed similarly well; data not presented). Note that these results are robust to the selection of the threshold for HSR (HSR >25%). Using a lower (for example) HSR threshold would affect both the proportion of positive cases and also the proportion of positive predictions, in contrast to examples in the literature where increases in prevalence are often associated with increases in sensitivity and decreases in precision (e.g., Leeflang et al., 2013).

As well as predictive accuracy, modeling allows us to assess the importance of different predictor variables for making predictions. The importance plot (Figure 3b) estimates the relative importance of variables in the multivariate models. The importance of variables varied slightly among the models, but air temperature, cumulative solar radiation, and minimum daily air temperature were important in most models, with wind speed being less useful. Bryant et al. (2023) came to a similar conclusion in developing the GHLI index, albeit wind speed was concluded to be of more importance than humidity.

Animal susceptibility to heat stress is known to vary with breed and age (Bryant et al., 2007) and including these as predictor variables in the current models (results not shown here) indicated a weak association. Compared with the variables reported in Figure 3b, they were generally the least important variables, and their inclusion improved model performance only slightly (sensitivity, precision, and F_1_ of cubist each improved by 3%–4%). Further work is required to explore their importance and how they could be incorporated into a generic prediction index.

Rumen bolus sensors were not primarily designed for heat stress detection, and the natural temperature cycles and buffering in the rumen make this challenging (Levit et al., 2021). However, by adjusting for cows' individual temperature variability we were able to detect extreme events on a group basis (proposed as >25% of animals having a rumen temperature greater than 3 SD above an individual animal's mean temperature). Using this definition allowed us to develop a predictive model for heat stress events that could be sufficiently sensitive and precise for operational management. The approach performed well for the purpose of proof of concept but needs to be verified by additional testing against independent datasets covering a greater range of environments. Uncertainty around this threshold is one reason why regression models rather than classification models were used for predicting heat stress events in this study. Classification models (such as logistic regression) would likely provide higher sensitivity and precision but assume certainty regarding which cases are positive or negative. Using regression models allowed us to adjust the HSR >25% threshold without refitting the models.

To develop a predictive model, the data were resampled to provide a training dataset that better balanced the number of positive and negative cases. Different resampling schemes could give different results and are another area for further investigation. In addition, the predictive models were trained using the default parameters in the caret package in R; improved performance might be obtained by further tuning. Finally, refinements to dataset construction could be made such as accounting for instances where some groups are HSR >25% and others are ≤25% at the same time and applying a differential weighting for group size.

Modeling results showed that linear heat indices such as GHLI and lm are not sufficiently discriminating for the purpose of heat stress prediction (at least as indicated by rumen temperature). However, nonlinear multivariate models such as cubist are potentially able to provide reliable and practical predictions of heat stress events, which would make them suitable for automated warning systems using forecast weather data.

## References

[bib1] Bar D., Kaim M., Flamenbaum I., Hanochi B., Toaff-Rosenstein R.L. (2019). *Technical note:* Accelerometer-based recording of heavy breathing in lactating and dry cows as an automated measure of heat load. J. Dairy Sci..

[bib2] Bewley J.M., Einstein M., Grott M.W., Schutz M. (2008). Comparison of reticular and rectal core body temperatures in lactating dairy cows. J. Dairy Sci..

[bib3] Boehmer B. (2015). Ruminal temperature as a measure of body temperature of beef cows and relationship with ambient temperature. Prof. Anim. Sci..

[bib4] Bohmanova J., Misztal I., Cole J.B. (2007). Temperature-humidity indices as indicators of milk production losses due to heat stress. J. Dairy Sci..

[bib5] Branco P., Torgo L., Ribeiro R.P. (2016). A survey of predictive modeling on imbalanced domains. ACM Comput. Surv..

[bib6] Bryant J.R., Huddart F., Schütz K.E. (2023). Development of a heat load index for grazing dairy cattle. N. Z. J. Agric. Res..

[bib7] Bryant J.R., López-Villalobos N., Pryce J.E., Holmes C.W., Johnson D.L. (2007). Quantifying the effect of thermal environment on production traits in three breeds of dairy cattle in New Zealand. N. Z. J. Agric. Res..

[bib8] Donkersloot, E. G., G. M. Worth, A. F. Yeates, M. D. Littlejohn, L. R. McNaughton, R. J. Spelman, and S. R. Davis. 2017. The benefit of a slick hair coat for heat tolerance in New Zealand dairy cattle. Pages 94–97 in Proc. Assoc. Adv. Anim. Breed Genet., Queensland, Australia.

[bib9] Grafarend E.W. (2006).

[bib10] Jago J., Beukes P., Cuttance E., Dalley D., Edwards J.P., Griffiths W., Saunders K., Shackleton L., Schütz K.E. (2023). Strategies to minimize the impact of climate change and weather variability on the welfare of dairy cattle in New Zealand and Australia. Anim. Prod. Sci..

[bib11] Kuhn M. (2008). Building predictive models in R using the caret package. J. Stat. Softw..

[bib12] Leeflang M.M., Rutjes A.W., Reitsma J.B., Hooft L., Bossuyt P.M. (2013). Variation of a test’s sensitivity and specificity with disease prevalence. CMAJ.

[bib13] Lees A.M., Lees J., Lisle A., Sullivan M., Gaughan J. (2018). Effect of heat stress on rumen temperature of three breeds of cattle. Int. J. Biometeorol..

[bib14] Levit H., Pinto S., Amon T., Gershon E., Kleinjan-Elazary A., Bloch V., Ben Meir Y.A., Portnik Y., Jacoby S., Arnin A., Miron J., Halachmi I. (2021). Dynamic cooling strategy based on individual animal response mitigated heat stress in dairy cows. Animal.

[bib15] Liu J.J., Li L.Q., Chen X.L., Lu Y.Q., Wang D. (2019). Effects of heat stress on body temperature, milk production, and reproduction in dairy cows: A novel idea for monitoring and evaluation of heat stress—A review. Asian-Australas. J. Anim. Sci..

[bib16] Molnar C., Bischl B., Casalicchio G. (2018). iml: An R package for interpretable machine learning. J. Open Source Softw..

[bib17] R Core Team (2023). https://www.R-project.org/.

[bib18] Thom E.C. (1959). The discomfort index. Weatherwise.

[bib19] Wickham H., Girlich M., Ruiz E. (2023). Dbplyr: A ‘dplyr’ Back End for Databases. [A computer program]. https://dbplyr.tidyverse.org/.

[bib20] Wijffels G., Sullivan M., Gaughan J. (2021). Methods to quantify heat stress in ruminants: Current status and future prospects. Methods.

[bib21] Wood S.N. (2011). Fast stable restricted maximum likelihood and marginal likelihood estimation of semiparametric generalized linear models. J. R. Stat. Soc. Series B Stat. Methodol..

